# Characteristics of Delayed Graft Function and Long-Term Outcomes After Kidney Transplantation From Brain-Dead Donors: A Single-Center and Multicenter Registry-Based Retrospective Study

**DOI:** 10.3389/ti.2024.12309

**Published:** 2024-03-01

**Authors:** Amanda Ahlmark, Ville Sallinen, Verner Eerola, Marko Lempinen, Ilkka Helanterä

**Affiliations:** Department of Transplantation and Liver Surgery, Abdominal Center, Helsinki University Hospital and University of Helsinki, Helsinki, Finland

**Keywords:** kidney donor profile index, long-term outcome, delayed graft function, kidney transplant, cold ischemia time

## Abstract

Delayed graft function (DGF) after kidney transplantation is common and associated with worse graft outcomes. However, little is known about factors affecting graft survival post-DGF. We studied the association of cold ischemia time (CIT) and Kidney Donor Profile Index (KDPI) with the long-term outcomes of deceased brain-dead donor kidneys with and without DGF. Data from Finland (*n* = 2,637) and from the US Scientific Registry of Transplant Recipients (SRTR) registry (*n* = 61,405) was used. The association of KDPI and CIT with the graft survival of kidneys with or without DGF was studied using multivariable models. 849 (32%) kidneys had DGF in the Finnish cohort. DGF and KDPI were independent risk factors for graft loss, [HR 1.32 (95% CI 1.14–1.53), *p* < 0.001, and HR 1.01 per one point (95% CI 1.01–1.01), *p* < 0.001, respectively], but CIT was not, [HR 1.00 per CIT hour (95% CI 0.99–1.02), *p* = 0.84]. The association of DGF remained similar regardless of CIT and KDPI. The US cohort had similar results, but the association of DGF was stronger with higher KDPI. In conclusion, DGF and KDPI, but not CIT, are independently associated with graft survival. The association of DGF with worse graft survival is consistent across different CITs but stronger among marginal donors.

## Graphical Abstract

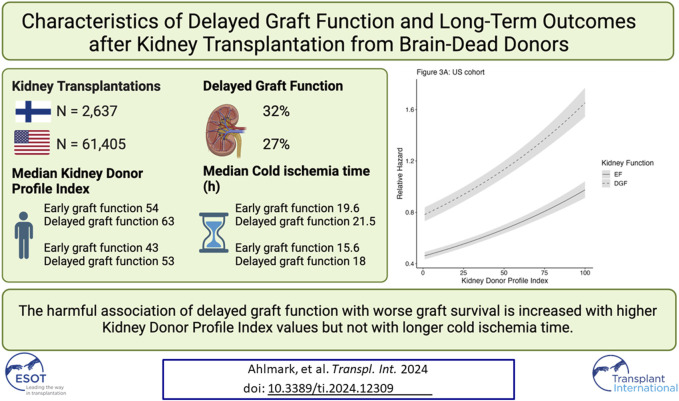

## Introduction

Delayed graft function (DGF) is still encountered in 20%–40% of all deceased donor kidney transplants, with higher frequencies being associated with expanded criteria donors [1–4]. DGF is considered to be the result of an ischemic-reperfusion injury, which arises during the procurement and subsequent cold storage of the graft as well as the reperfusion during implantation [5, 6]. DGF has been linked to worse graft survival rates [6, 7] and higher rates of acute rejection [6], although contradicting results also exist [8]. A meta-analysis found increased risk of graft failure, acute rejection, and mortality associated with DGF [9]. The most significant risk factors for DGF are increased donor age, increased kidney donor profile index (KDPI), and increased cold ischemia time (CIT) [3, 10–13].

The increasing demand for kidneys and the growing use of extended criteria kidneys underscores the importance of understanding the complex nature of DGF and factors affecting the long-term outcomes of kidneys with DGF, as the rate of DGF is reportedly increasing over time [3]. However, conclusive evidence on factors affecting the long-term outcomes among kidney transplants with DGF is still lacking. Furthermore, as most studies have a regional cohort that affects both donor and recipient characteristics, universal conclusions are difficult to reach. While the effect of acute rejection might have little cumulative effect on the outcomes of kidneys with DGF [14], it remains unclear whether the association of DGF with graft survival is similar among patients with longer CIT or higher KDPI. Some transplant programs, such as the Eurotransplant senior program, aim to minimize CIT among older kidney donors. It has been suggested that longer CIT would be more harmful in older donor kidneys or kidneys with poor quality [15], especially due to the occurrence of DGF. However, our recent study suggested that the effect of longer CIT is not more harmful among older donors or donors with high KDPI [16]. The role of pretransplantation biopsies has also been discussed in literature. The histologic findings might affect the allocation process, and a single-center study found that both the rate of DGF was higher, and the graft survival was worse among kidneys with a suboptimal histological score [17].

This study aims to examine the association of DGF with graft survival using a national cohort from Finland and to study whether the association of DGF with graft survival differs in subgroups based on KDPI and CIT. Furthermore, the aim is to confirm these findings in a larger US cohort using data from the Scientific Registry of Transplant Recipients (SRTR).

## Materials and Methods

### Study Population and Data Collection

This study was a retrospective observational registry analysis. The initial study population consisted of all adult (age >16 years) patients receiving deceased donor kidney transplants performed at Helsinki University Hospital (HUH), Finland from 12 May 2004 to 31 December 2019. HUH is the only transplantation center in Finland. Patients with primary nonfunction or graft loss within the first week after transplantation (*n* = 73, 2%) were excluded. In addition, living donor kidneys, pediatric recipients (age <16 years), and recipients of multiorgan transplants (total *n* = 565, 17%) were excluded. All donors were brain-dead donors, as donation after circulatory death (DCD) was not implemented in Finland during the study period. Machine perfusion was not used in Finland during the study period. Due to the definition of DGF (need for dialysis during the first post-transplant week), and because patients were not accepted to the waiting list pre-emptively in Finland during the study period, only patients who were on dialysis pretransplantation were included. Patients and their pre-and post-transplant data were collected from the Finnish Kidney Transplant Registry, which is a national registry for the follow-up of kidney transplant patients obliged by law. Patients were followed until death, graft loss, or 31 December 2020.

In addition, this study used data from the Scientific Registry of Transplant Recipients (SRTR). The SRTR data system includes data on all donors, waitlisted candidates, and transplant recipients in the United States, submitted by the members of the Organ Procurement and Transplantation Network (OPTN). The Health Resources and Services Administration, US Department of Health and Human Services, provides oversight to the activities of the OPTN and SRTR contractors. To create a dataset similar to the Finnish data, only deceased brain-dead donor kidney-only transplant recipients between 01 January 2014 and 09 September 2019 with pretransplant dialysis treatment were included, i.e., donation after circulatory death (DCD) kidneys were excluded. Patients were followed until death, graft loss, or 9 September 2020.

Cases with missing data were excluded from the analyses, due to the low number of missing data (<3% in both cohorts).

This study was approved by the institutional review board of Helsinki University Hospital (HUS/115/2020) and SRTR. The clinical and research activities being reported are consistent with the Principles of the Declaration of Istanbul as outlined in the Declaration of Istanbul on Organ Trafficking and Transplant Tourism.

### Definitions

DGF was defined as the need for dialysis during the first seven postoperative days [18].

KDPI was calculated as described on the Organ Procurement and Transplantation Network website [19]. The KDPI values were calculated using 2019 KDPI reference values. For donors with unknown status of diabetes and/or hypertension, KDPI was calculated as instructed on the OPTN website [19].

### Statistical Analyses

Categorical variables are described as the number of cases and percentages. As the distributions within either dataset were not normal, continuous variables are described as median and interquartile range. Mann-Whitney-U-test was used to assess statistical significance for differences in the continuous variables and Chi Square test was used for categorical data.

Kaplan-Meier survival curves were used to analyze graft survival, with both death with functioning graft and return to dialysis as outcomes. Differences between the studied groups were analyzed with the log-rank test. Multivariable Cox regression analysis was used to examine risk factors for graft loss. Sensitivity analyses were performed using death-censored graft loss as the binary outcome. Two-sided *p*-values <0.05 were considered statistically significant. Variables chosen for the multivariable model were earlier confirmed risk factors for DGF or variables significant in univariable models. When KDPI was included in the models, all the other donor factors used to calculate KDPI were left out of the model due to possible multi-collinearity (age, race, body mass index (BMI), history of hypertension, history of diabetes, and cause of death). Interactions between CIT and DGF as well as KDPI and DGF were used to analyze whether the risk associated with DGF differed according to cold ischemia time or KDPI value. To account for clustered data due to the relationship between kidneys from the same donor, the Huber-White method served to adjust the standard errors of the regression coefficients and provide robust standard errors of the coefficients [20].

We assessed the validity of the Cox model by plotting the scaled Schoenfeld residuals for testing the proportional hazards assumption, using visualization of deviance residuals for checking influential outliers and testing for non-linearity. Restricted cubic splines were used to determine the nonlinearity of the associations and for plotting nonlinear associations between covariates and the outcome, as regression models require the assumption of linearity. Variables plotted by restricted cubic splines are reported as figures and *p*-values, and other variables are reported as hazard ratios with 95% confidence intervals.

Statistical analysis was performed using R (R Core Team, 2023), RStudio (Posit Team, 2023), and the R packages survival (Thernau, 2023), survminer (Kassambara, Kosinski, Biecek, 2021), ggplot2 (Wickham, 2016), gtsummary (Sjoberg, Whiting, Curry, Labery, Larmarange. 2021), and rms (Harrell, 2023).

## Results

### Finnish Study Population

A total of 3,275 kidney transplants were performed during the period 12 May 2004 to 31 December 2019 in Finland. After excluding grafts that were lost during the first week, pediatric recipients, living donor kidney recipients, and recipients of multiorgan transplants, the final study population consisted of a total of 2,637 patients receiving kidney transplants, of which 865 (32%) had DGF. Demographic characteristics of the Finnish study population grouped by early function (EF) and DGF are presented in Table 1. DGF was more frequent among male recipients, recipients receiving kidneys from male donors, older recipients, and recipients receiving kidneys from older donors. KDPI was higher and CIT was longer among recipients with DGF. Recipients with DGF more often had one or several previous kidney transplants compared to recipients with EF.

**TABLE 1:** Finnish cohort characteristics.

Variable	Kidney function		
	EF	DGF	*p*-value^b^
	N = 1,788 (68%)^a^	N = 849 (32%)^a^	
Donor age (years)	55 (44,64)	58 (50,65)	<0.001
Donor sex			0.019
Female	817 (46%)	346 (41%)	
Male	971 (54%)	503 (59%)	
Kidney Donor Profile Index	54.0 (31.0, 77.0)	63.0 (44.0, 82.0)	<0.001
Cold ischemia time (hours)	19.6 (16.2, 22.8)	21.5 (18.3, 24.3)	<0.001
HLA mismatch	3.0 (2.0, 3.0)	3.0 (2.0, 3.0)	0.282
Recipient age at transplant (years)	54 (43,62)	55 (46,63)	0.002
Recipient sex			0.013
Female	665 (37%)	273 (32%)	
Male	1,123 (63%)	576 (68%)	
Recipient diabetes	407 (23%)	220 (26%)	0.084
Recipient previous kidney transplant	182 (10%)	134 (16%)	<0.001
Recipient peak PRA	0.0 (0.0, 20.0)	0.0 (0.0,33.0)	0.001
Follow-up time (months)	61.3 (28.3,114.3)	73.7 (39.3,109.8)	0.004
Donor cause of death			<0.001
Anoxia	61 (3%)	18 (2%)	
Cerebral hemorrhage	1,027 (56%)	495 (57%)	
Stroke	64 (3%)	50 (6%)	
Trauma	432 (23%)	167 (19%)	
Other	258 (14%)	137 (16%)	

^a^ Median (25%, 75%); n (%).

^b^ Mann-Whitney test for continuous, Chi-square for categorical variables EF, early function; DGF, delayed graft function; HLA, human leukocyte antigens; PRA, panel reactive antibodies.

### Early Function vs. Delayed Graft Function in Finland

**FIGURE 1:**
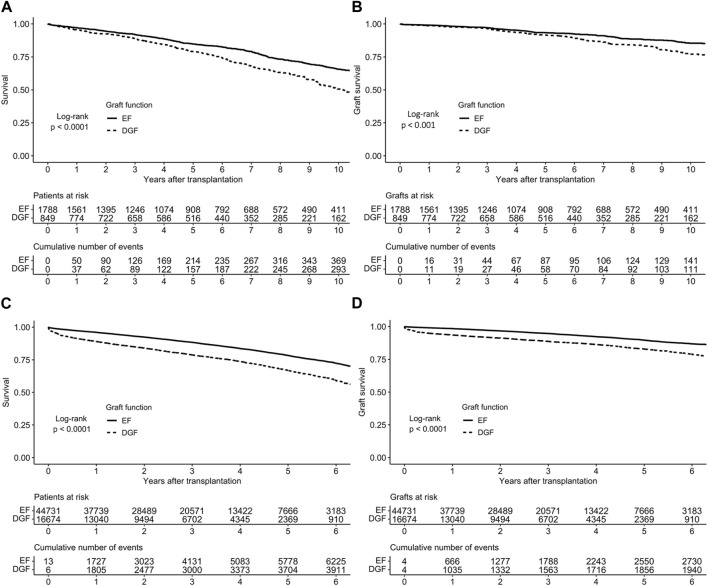
Survival of early and delayed graft function kidney transplants in **(A)** the Finnish cohort and **(C)** the US cohort and death-censored survival in **(B)** the Finnish cohort **(D)** the US cohort, based on Kaplan-Meier estimates.

Graft survival estimates were significantly lower among recipients with DGF compared to recipients with EF in unadjusted analyses (*p* < 0.001, Figure 1A), with 10-year survival among patients with EF being 66% (CI 95% 63%–69%), and 51% (CI 95% 46%–55%) among patients with DGF. Additionally, the hazard ratio (HR) for graft loss or death for DGF in the univariable analysis was 1.53 (CI 95% 1.33–1.77, *p* < 0.001). In multivariable analysis (adjusted for CIT, KDPI, peak PRA >30%, previous kidney transplant, recipient age and sex, and recipient pre-transplant diabetes as well as accounting for clustering), DGF was independently associated with worse graft survival (HR 1.32, 95% CI 1.14–1.53; Table 2). The unadjusted analyses were also performed with death-censored graft loss as outcome, and the results remained similar (Figure 1B).

**TABLE 2:** Multivariable Cox regression results for time to graft loss, Finnish cohort (N = 2,637).

Characteristic	HR	95% CI	*p*-value
Delayed graft function	1.32	1.14, 1.53	<0.001
Cold ischemia time (per hour)	1.00	0.99, 1.02	0.84
Kidney Donor Profile Index (per one point)	1.01	1.01, 1.01	<.001
Recipient diabetes	1.96	1.69, 2.28	<0.001
Recipient age (per year)	Not applicable^a^		0.015^b^
Recipient sex (male)	1.17	1.00, 1.36	0.05
Recipient peak PRA over 30%	1.08	0.86, 1.35	0.52
Previous kidney transplant	1.61	1.24, 2.08	<0.001

HR, hazard ratio; CI, confidence interval; PRA, panel reactive antibodies.

^a^ Not applicable due to non-linearity.

^b^ *p*-value for non-linearity.

In the Finnish cohort, all variables met the proportional hazards assumption, and the associations of all continuous variables were linear except for recipient age. The association of KDPI was plotted as non-linear, even if the non-linearity *p*-value was non-significant, as the plotted model visualizes the association of KDPI better than an HR value.

### Cold Ischemia Time in Finland

**FIGURE 2:**
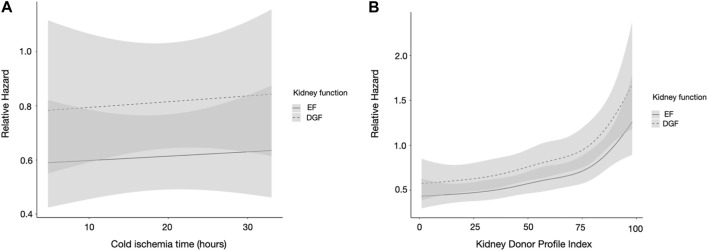
**(A,B)** Restricted cubic spline analysis of **(A)** cold ischemia time and **(B)** kidney donor profile index and the association of kidney function, in the Finnish cohort. DGF is portrayed by the dashed line and EF by the solid line. CIs are portrayed in light gray, overlapping CIs in dark gray. The model is adjusted for CIT, recipient age, sex, recipient diabetes, peak PRA, and previous kidney transplant. CIT, cold ischemia time; PRA, panel reactive antibodies; KT, kidney transplant; DGF, delayed graft function; EF, early function; CI, confidence interval.

Longer CITs were not independently associated with worse graft survival in multivariable analysis (Table 2). In a plotted association of DGF with graft survival, the association remained similar regardless of CIT (Figure 2A). There was no significant interaction between CIT and DGF, *p* = 0.824. The survival rates of EF and DGF kidneys with CITs longer and shorter than 18 h can be found in Supplementary Table S1.

### Kidney Donor Profile Index in Finland

KDPI was an independent risk factor for graft loss in multivariable analyses (Table 2). In a plotted prediction of the association of DGF with graft survival, the difference between EF and DGF kidneys remained similar regardless of KDPI value. The association of higher KDPI was similar in both graft function groups (Figure 2B). There was no significant interaction between KDPI and DGF, *p* = 0.217. DGF kidneys with KDPI values ≥85 had the worst survival rates, while EF kidneys with KDPI ≥85 and DGF kidneys with KDPI <85 had similar survival rates; the survival rates of EF and DGF kidneys based on KDPI can be found in Supplementary Table S1.

### Validations of Results With SRTR Data

Altogether, 94,154 kidney-only transplantations were performed from deceased donors in the US between 01 January 2014 and 09 September 2019. From these the following groups were excluded: pre-emptive transplantations (*n* = 10,782), <20 years old (*n* = 3,812), primary non-function (*n* = 312), DCD donors (*n* = 17,840) and cases with missing data (*n* = 3), resulting in a final cohort of 61,404 kidney transplantations. The characteristics of the SRTR cohort are presented in Table 3.

**TABLE 3:** US cohort characteristics.

Variable	Kidney function		
	EF	DGF	*p*-value^b^
	N = 44,731 (73%)^a^	N = 26,674 (27%)^a^	
Donor age (years)	37 (25,50)	42 (30,53)	<0.001
Donor sex			0.034
Female	18,030 (40%)	6,563 (39%)	
Male	26,701 (60%)	10,111 (61%)	
Kidney Donor Profile Index	43.0 (22.0, 66.0)	53.0 (33.0, 73.0)	<0.001
Cold ischemia time (hours)	15.6 (10.4, 21.5)	18.0 (12.4, 24.2)	<0.001
HLA mismatch	4.0 (3.0, 5.0)	4.0 (4.0, 5.0)	<0.001
Recipient age at transplant (years)	54 (42,63)	56 (46,64)	<0.001
Recipient sex			<0.001
Female	18,728 (42%)	5,431 (33%)	
Male	26,003 (58%)	11,243 (67%)	
Recipient diabetes	15,152 (34%)	7,252 (44%)	<0.001
Recipient previous kidney transplant	5,883 (13%)	2,233 (13%)	0.442
Follow up time (months)	35.2 (17.7,53.2)	29.4 (12.3,48.2)	<0.001
Donor cause of death			<0.001
Anoxia	17,378 (39%)	7,064 (42%)	
Cerebrovascular/stroke	11,443 (26%)	5,179 (31%)	
Head trauma	14,648 (33%)	3,998 (24%)	
CNS Tumor	207 (0%)	62 (0%)	
Other	1,055 (2%)	371 (2%)	
Donor serum creatinine	0.9 (0.7–1.3)	1.2 (0.8–2.2)	<0.001
Donor ethnicity			<0.001
Asian	1,114 (2%)	519 (3%)	
Black	7,255 (16%)	2,639 (16%)	
Multi	175 (0%)	85 (1%)	
Native	284 (1%)	89 (1%)	
Pacific	157 (0%)	62 (0%)	
White	35,756 (80%)	13,280 (80%)	
Recipient ethnicity			<0.001
Asian	3,318 (7%)	1,239 (7%)	
Black	15,399 (34%)	6,653 (40%)	
Multi	337 (1%)	115 (1%)	
Native	436 (1%)	205 (1%)	
Pacific	236 (1%)	83 (0%)	
White	25,005 (56%)	8,379 (50%)	

^a^ Median (25%, 75%); n (%).

^b^ Mann-Whitney test for continuous, Chi-square for categorical variables EF, early function; DGF, delayed graft function; HLA, human leukocyte antigens.

DGF occurred in 26,674 recipients (27%). Graft survival estimates were significantly lower among recipients with DGF compared to recipients with EF in unadjusted analyses (Figures 1C, D). In a multivariable model, (adjusted for CIT, KDPI, previous kidney transplant, recipient sex, recipient age, recipient diabetes and use of machine perfusion), DGF was an independent risk factor for graft loss (HR 1.63, 95% CI 1.48–1.80; Table 4).

**TABLE 4:** Multivariable Cox regression results for time to graft loss, US cohort (N = 60,919).

Characteristic	HR	95% CI	*p*-value
Delayed graft function	1.63	1.48, 1.80	<0.001
Cold ischemia time (per hour)	1.00	1.00, 1.00	0.228
Kidney donor profile index (per one point)	Not applicable^a^		< 0.001^b^
Recipient diabetes	1.29	1.24, 1.35	<0.001
Recipient age (per year)	Not applicable^a^		<0.001^b^
Recipient sex (male)	1.10	1.05, 1.14	<0.001
Machine perfusion	1.08	1.04, 1.12	<0.001
Previous kidney transplant	1.14	1.08, 1.21	<0.001

HR, hazard ratio; CI, confidence interval.

^a^ Not applicable due to non-linearity.

^b^ *p*-value for non-linearity.

The association of recipient age were nonlinear and thus modeled with restricted cubic splines. Other continuous variable associations were linear.

**FIGURE 3:**
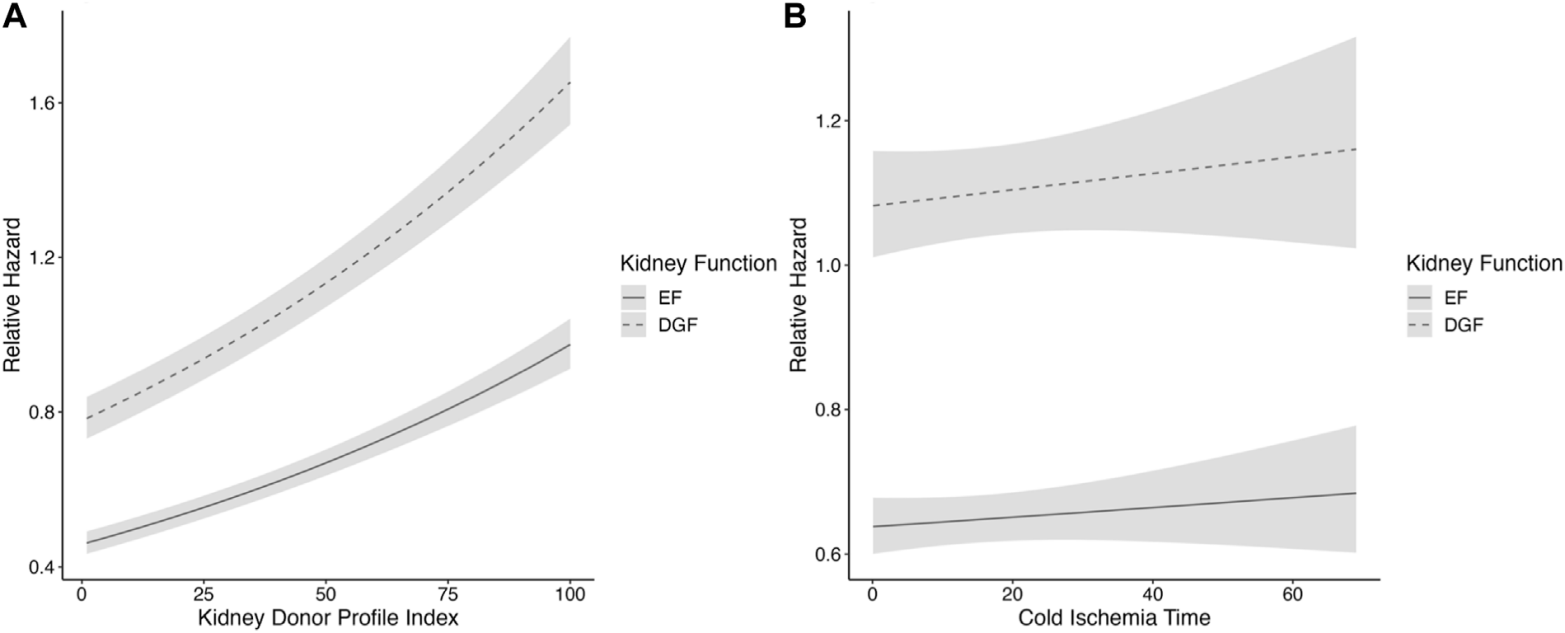
**(A,B)** Restricted cubic spline analysis of **(A)** kidney profile index and **(B)** cold ischemia time and the association of kidney function, in the SRTR cohort. DGF is portrayed by the dashed line and EF by the solid line. CIs are portrayed in light gray. The model is adjusted for CIT, recipient age, sex, recipient diabetes, machine perfusion, and previous kidney transplant. CIT, cold ischemia time; PRA, panel reactive antibodies; KT, kidney transplant; DGF, delayed graft function; EF, early function; CI, confidence interval.

As there was a violation of the proportionality assumption of the onset of graft function (*p* < 0.001), CIT (*p* = 0.01) and recipient diabetes (*p* = 0.007), Schoenfeld residuals and Kaplan-Meier curve were assessed and deemed acceptable; the plotted Schoenfeld residuals can be found in Supplementary Figure S1. The association of KDPI with worse graft survival was stronger in DGF kidneys with high KDPI values, compared to EF kidneys (Figure 3A). There were no significant interactions between CIT and DGF (*p* = 0.051), KDPI and DGF (*p* = 0.571) or machine perfusion and DGF (*p* = 0.814). Although linear, the associations of KDPI and CIT on graft survival were also plotted as the plotted model visualizes the associations better than an HR value (Figures 3A, B).

Survival rates of EF and DGF kidneys in the US cohort based on KDPI values and CIT can be found in Supplementary Table S2.

### Death-Censored Graft Survival Analyses

Multivariable regression analyses were also performed with death-censored graft loss as the outcome, and the results remained similar in the Finnish cohort (Supplementary Table S3; Figure 2), which suggests that other causes of death are not large confounders. Similar results were found in the SRTR cohort (Supplementary Table S4; Supplementary Figures S3, S4).

### Regression Model Based on Time-Splitting

A time-splitting was also made because of the violations of the proportional hazards assumption in the US cohort, and the associations of CIT and KDPI on graft survival were assessed during the first follow-up year as well as the time after the first year separately. No large differences were found (Supplementary Figures S5–S8).

## Discussion

In our study, we found that DGF kidneys have worse graft survival compared to EF kidneys, as expected. The harmful association of DGF with graft survival remained similar regardless of CIT length. This, along with the non-significant interactions between CIT and DGF, suggests that the harmful effect of DGF is not increased when CIT increases. These findings are also supported by an earlier study [21].

KDPI was found to be an independent risk factor for graft loss in multivariable analyses, as expected. In plotted predictions of the association of DGF with graft survival as a function of KDPI, the association of DGF remained similar. No significant interaction between DGF and KDPI could be found in the Finnish cohort.

The findings of the US cohort support the findings from the Finnish cohort that the risk of graft loss associated with DGF is similar in various CIT lengths. However, in the US cohort, the association of DGF on graft survival as a function of KDPI shows a stronger association of DGF with high KDPI. No significant interaction between DGF and KDPI could be found. The difference seen is not as noticeable in the Finnish cohort, which partly could be explained by the smaller cohort.

Many transplanted kidneys to this day still suffer from DGF and thus it is essential that the causes of DGF are understood and that routines to minimize other factors affecting the long-term outcomes, such as acute rejection, are used. With a greater understanding of the process of DGF, transplantation procedures, and pre- and post-operative care can be planned most beneficially.

Previous studies have concluded that DGF is associated with worse graft survival [6, 7] and increased mortality [9, 22]. In both our cohorts we recorded worse graft survival for DGF kidneys.

Longer CIT has been identified as a risk factor for DGF [3, 10, 11, 16], and increased CIT has also been associated with higher risks for graft failure in some studies [23, 24], but not in others [21]. In our current study, CIT was not an independent risk factor for graft loss. The differences between study results remains somewhat unclear, possibly different analytical strategies (CIT as a continuous variable or categorized) might explain some of the discrepancies. However, as CIT has been recognized as a risk factor for DGF, protocols designed to reduce the CIT are also beneficial to reduce the risk of graft loss, as well as the size of costs and length of hospital stays, since DGF has been associated with poor graft survival, higher costs, and longer hospital stays [25]. One study also described DGF leading to a more complex post-operative course for the patient [6].

Higher KDPI values have been associated with increased risk for DGF [3, 10–12]. The effects of KDPI on graft survival have been studied using mixed cohorts of both EF and DGF kidneys, and a few studies could be found where the impact of KDPI had been studied on a DGF population [7, 26]. One of these studies showed that KDPI >85% was associated with worse outcomes in both EF and DGF kidneys which was confirmed in our study of a larger cohort [7]. As donors with KDPI >85% have been compared to the earlier used designation extended criteria donors [12], a worse graft survival estimate of these kidneys is in line with earlier research. Another study showed increased risk of graft loss in DGF kidneys with kidney donor risk index >1 [26]. One study examined the risk of DGF and graft loss in standard vs. extended criteria donors from both brain-dead donors and DCD donors. This study found that the DGF risk was increased in extended criteria DCD donations compared to standard criteria DBD donations, but did not find a difference in the risk of graft loss in any group compared to standard criteria DBD donations [27].

As the study is retrospective, there are several limitations to the study. Data that was missing was excluded, instead of using imputation, as the number of cases with missing data was very low. The Finnish cohort is smaller than the US cohort, which could lead to a risk of under-powered results in the Finnish cohort. We chose to include two different cohorts for better generalizability, as studies have shown that differences exist in graft survival between different countries [28–30], and also the deceased donor characteristics are different between the US and Europe, with older donors increasingly utilized in Europe. Furthermore, relating to the retrospective nature of the study, the cause and effect cannot be proven, and only associations between DGF and CIT as well as KDPI could be shown. Efforts to minimize potential bias and confounding were made, by using two different cohorts as well as analyzing the data with multivariable regression models. The associations studied are complex, and DGF is not a clean confounder and can work as a mediator as well. Using a large cohort helps with both minimizing bias and confounding. Graphical visualization aids in showcasing these complex associations. In our study we focused on DBD kidneys as they are still the majority of transplantations, but it is also noteworthy that DCD donations are increasing in clinical practice and the risk of DGF is much higher among DCD kidneys. Studying and understanding the risks regarding DGF and graft survival in DCD kidney transplantation would be highly important in the future.

Since the increasing demand for kidneys drives allocation processes to use extended criteria donors, knowledge of potential increased risks is important. Although higher KDPI values are associated with a greater risk for graft loss, the risk of graft loss associated with DGF remained similar in a wide range of KDPI values in our study, suggesting that other aspects of the transplantation process play a role in the long-term outcomes of kidney transplants as well.

In conclusion, our study shows that the association of DGF with graft survival does not change with CIT length and that the association of DGF is higher among kidneys with higher KDPI values.

To meet the future demand for kidneys and make the most of the available kidneys in the allocation process, further knowledge of the nature of DGF and factors affecting the long-term outcomes of kidneys with DGF is necessary. For example, understanding the histological and molecular changes in kidneys with DGF could help in understanding the risks following DGF, and could further facilitate the use of marginal kidneys for the benefit of wait-listed patients.

## Data Availability

The data analyzed in this study is subject to the following licenses/restrictions: Access to the Finnish datasets is not readily available as it is limited by national regulations and restrictions regarding sharing transplant patient data. Access to the US dataset is subject to limitations outlined in the current data use agreements with SRTR. Requests to access these datasets should be directed to SRTR https://www.srtr.org/contact-us/contact-form/.

## Abbreviation

DBD, donation after brain death; DCD, donation after circulatory death; DGF, delayed graft function; EF, early function; CIT, cold ischemia time; KDPI, Kidney Donor Profile Index; HLA-MM, human leukocyte antigen mismatch; PRA, panel reactive antibodies; SRTR, Scientific Registry of Transplant Recipients.
