# Supplementary material for: Characteristics of Delayed Graft Function and Long-Term Outcomes After Kidney Transplantation From Brain-Dead Donors: A Single-Center and Multicenter Registry-Based Retrospective Study
Source: Transpl Int. 2024 Mar 1;37:12309. doi: 10.3389/ti.2024.12309 (PMC10942003; doi:10.3389/ti.2024.12309)
Supplement: Supplementary file 1 [file DataSheet1.pdf]

Table of Contents

Supplemental material **2**

Capsule summary **2**

Supplemental figures **2**

Supplemental figure 1 2
Supplemental figure 2 3
Supplemental figure 3 4
Supplemental figure 4 5
Supplemental figure 5 6
Supplemental figure 6 7
Supplemental figure 7 8
Supplemental figure 8 9

Supplemental tables **9**

Supplemental table 1 9
Supplemental table 2 10
Supplemental table 3 10
Supplemental table 4 11

Supplemental material

Capsule summary

The harmful association of delayed graft function with worse graft survival is increased with higher Kidney Donor Profile Index values but not with longer cold ischemia time.

Supplemental figures

Supplemental figure 1

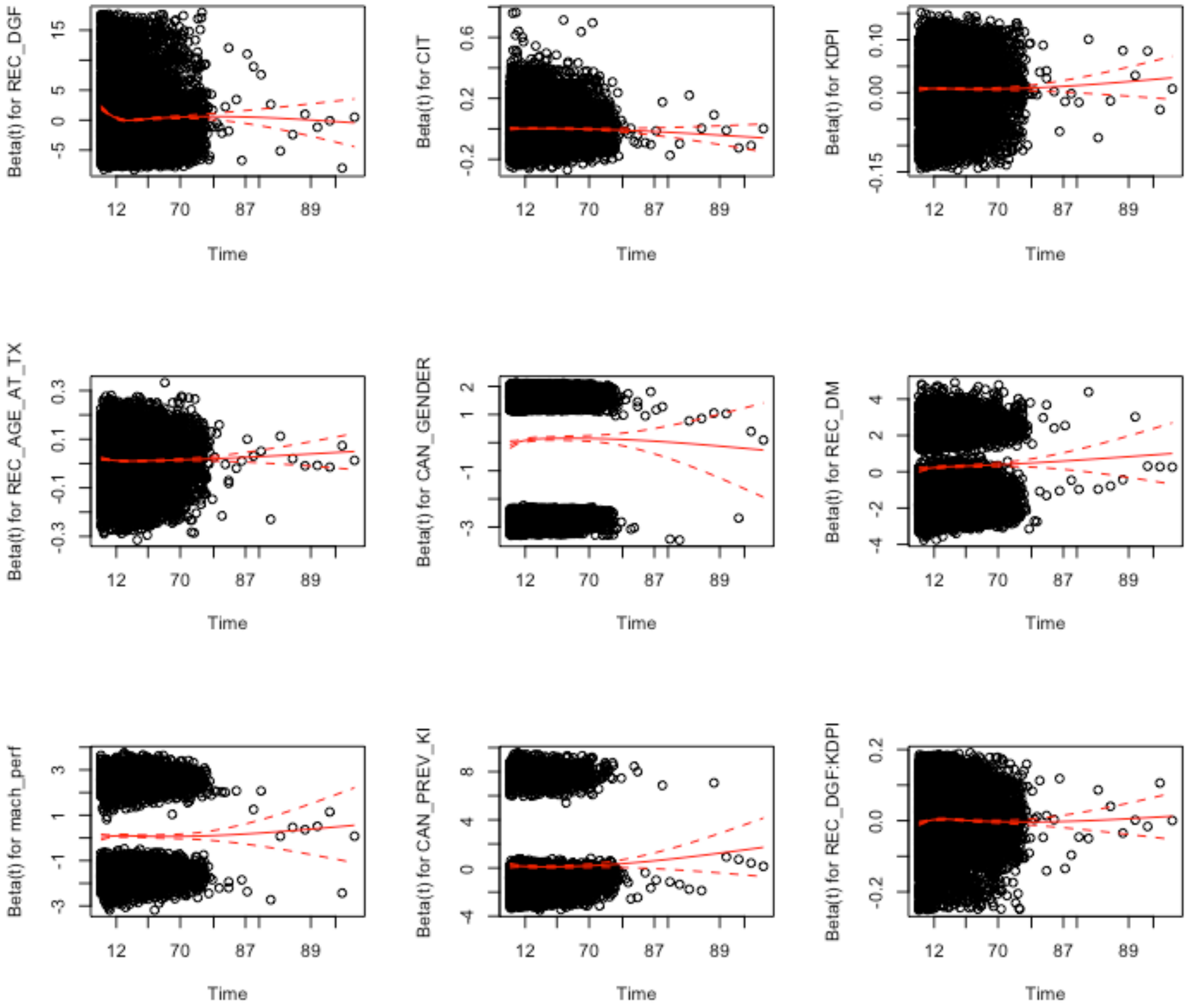

Supplemental figure 1
Schoenfeld residuals from multivariable cox regression of graft survival, US cohort

Supplemental figure 2

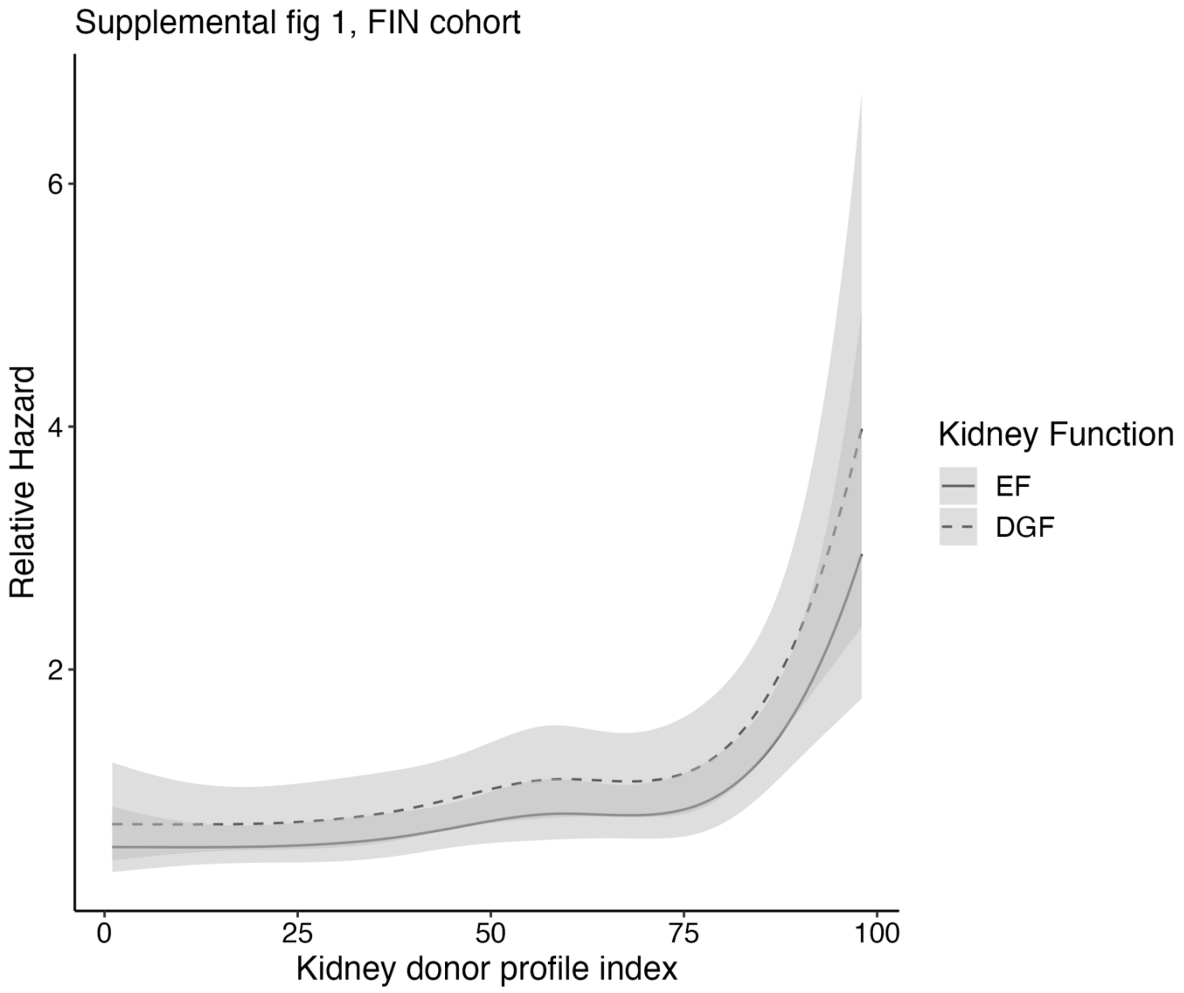

Supplemental figure 2
Restricted cubic spline analysis of kidney donor profile index as well as the association of kidney function, with death censored graft loss, in the Finnish cohort. DGF is portrayed by the yellow line and EF by the black line. The model is adjusted to CIT, recipient age, sex, recipient diabetes, machine perfusion, previous kidney transplant.
CIT = cold ischemia time, KT = kidney transplant, DGF = delayed graft function, EF = early function

Supplemental figure 3

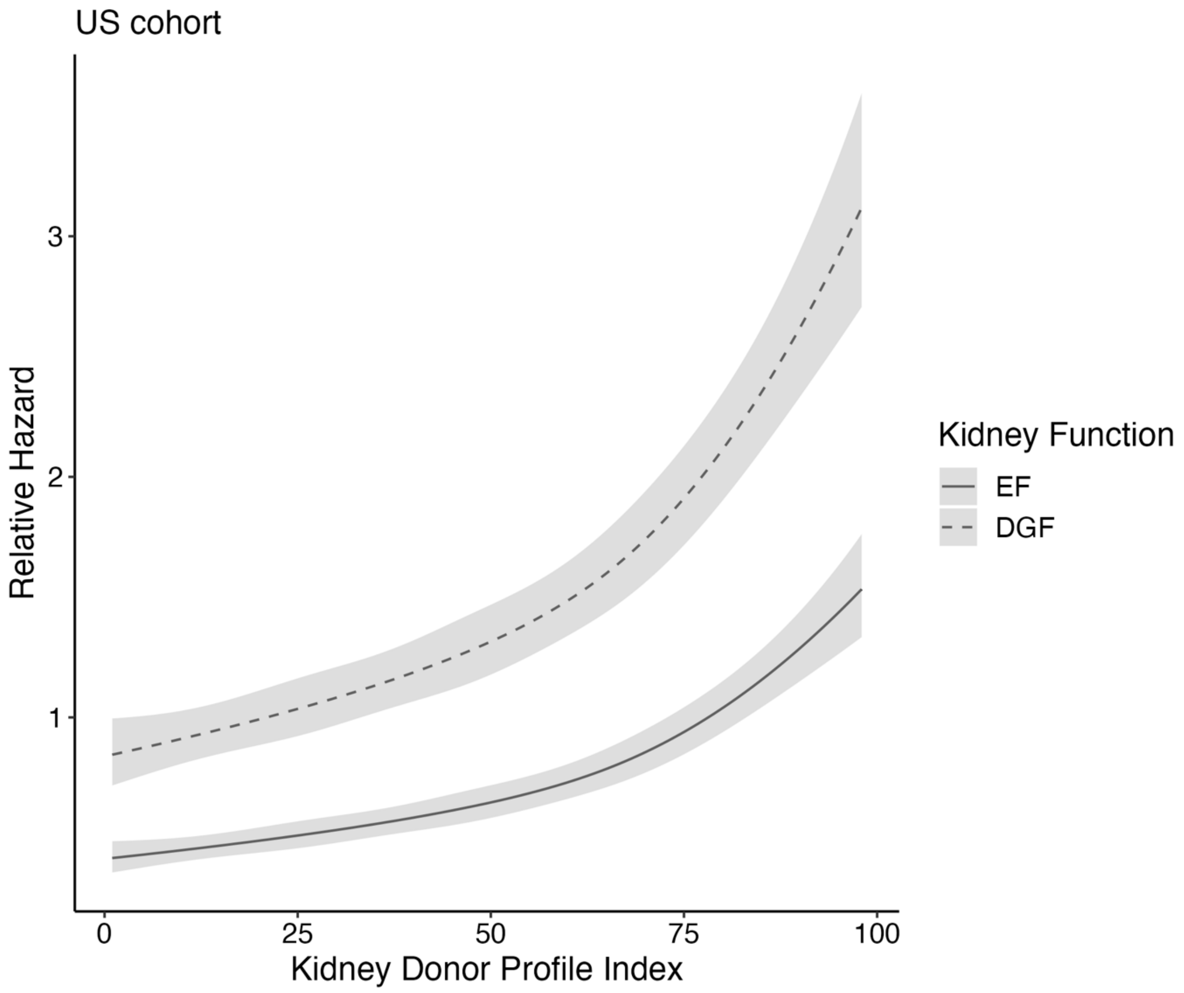

Supplemental figure 3 Restricted cubic spline analysis of kidney donor profile index as well as the association of kidney function, with death censored graft loss, in the US cohort. DGF is portrayed by the dashed line and EF by the solid line. CIs are portrayed in light gray. The model is adjusted to CIT, recipient age, sex, recipient diabetes, machine perfusion, previous kidney transplant.

CIT = cold ischemia time, KT = kidney transplant, DGF = delayed graft function, EF = early function

Supplemental figure 4

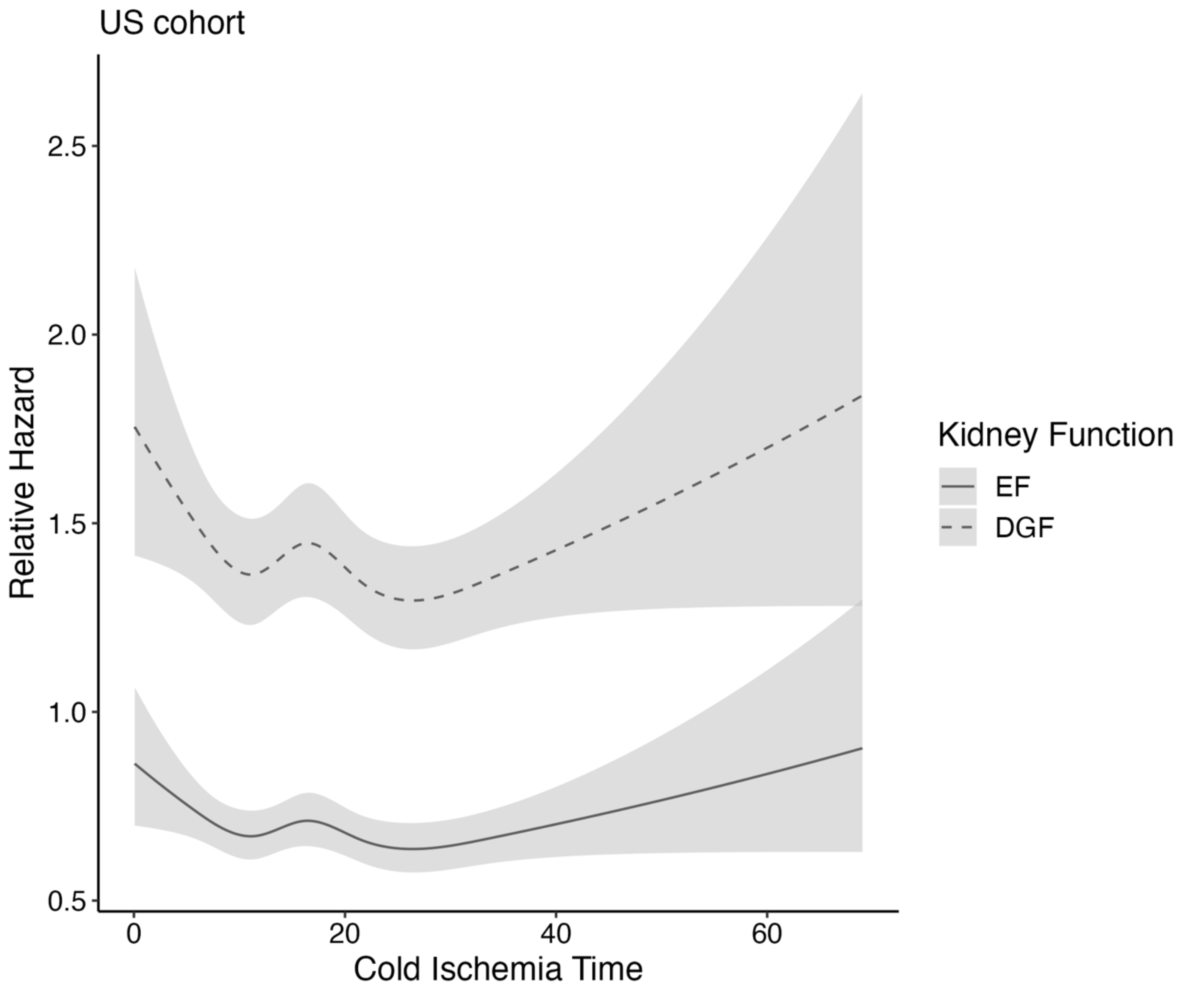

Supplemental figure 4
Restricted cubic spline analysis of cold ischemia time as well as the association of kidney function, with death censored graft loss, in the US cohort. DGF is portrayed by the dashed line and EF by the solid line. CIs are portrayed in light gray. The model is adjusted to CIT, recipient age, sex, recipient diabetes, machine perfusion, previous kidney transplant.
CIT = cold ischemia time, KT = kidney transplant, DGF = delayed graft function, EF = early function

Supplemental figure 5

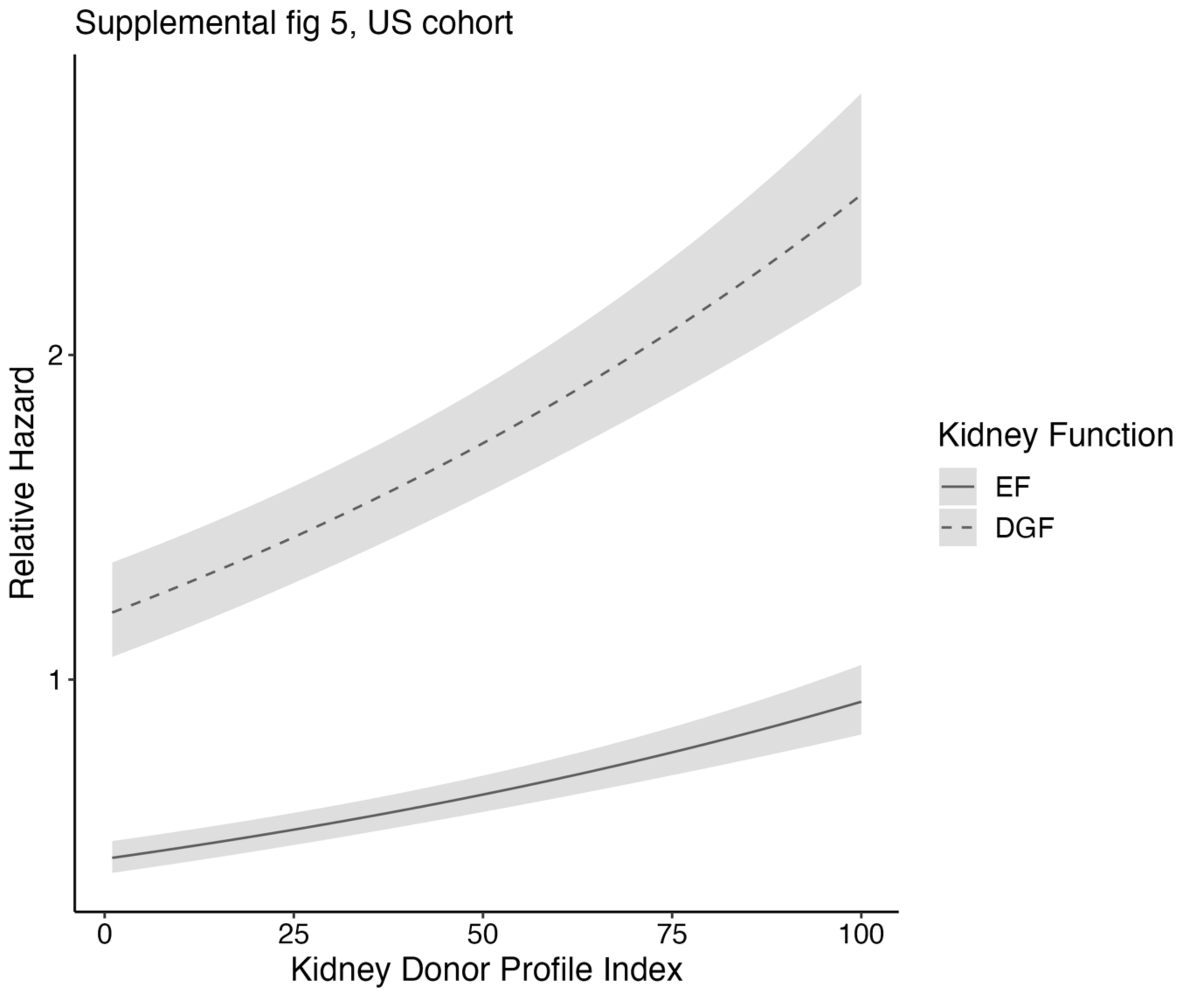

Supplemental figure 5

Restricted cubic spline analysis of kidney donor profile index as well as the association of kidney function, during the first follow up year, in the US cohort. DGF is portrayed by the dashed line and EF by the solid line. The model is adjusted to CIT, recipient age, sex, recipient diabetes, machine perfusion, previous kidney transplant.

CIT = cold ischemia time, KT = kidney transplant, DGF = delayed graft function, EF = early function

Supplemental figure 6

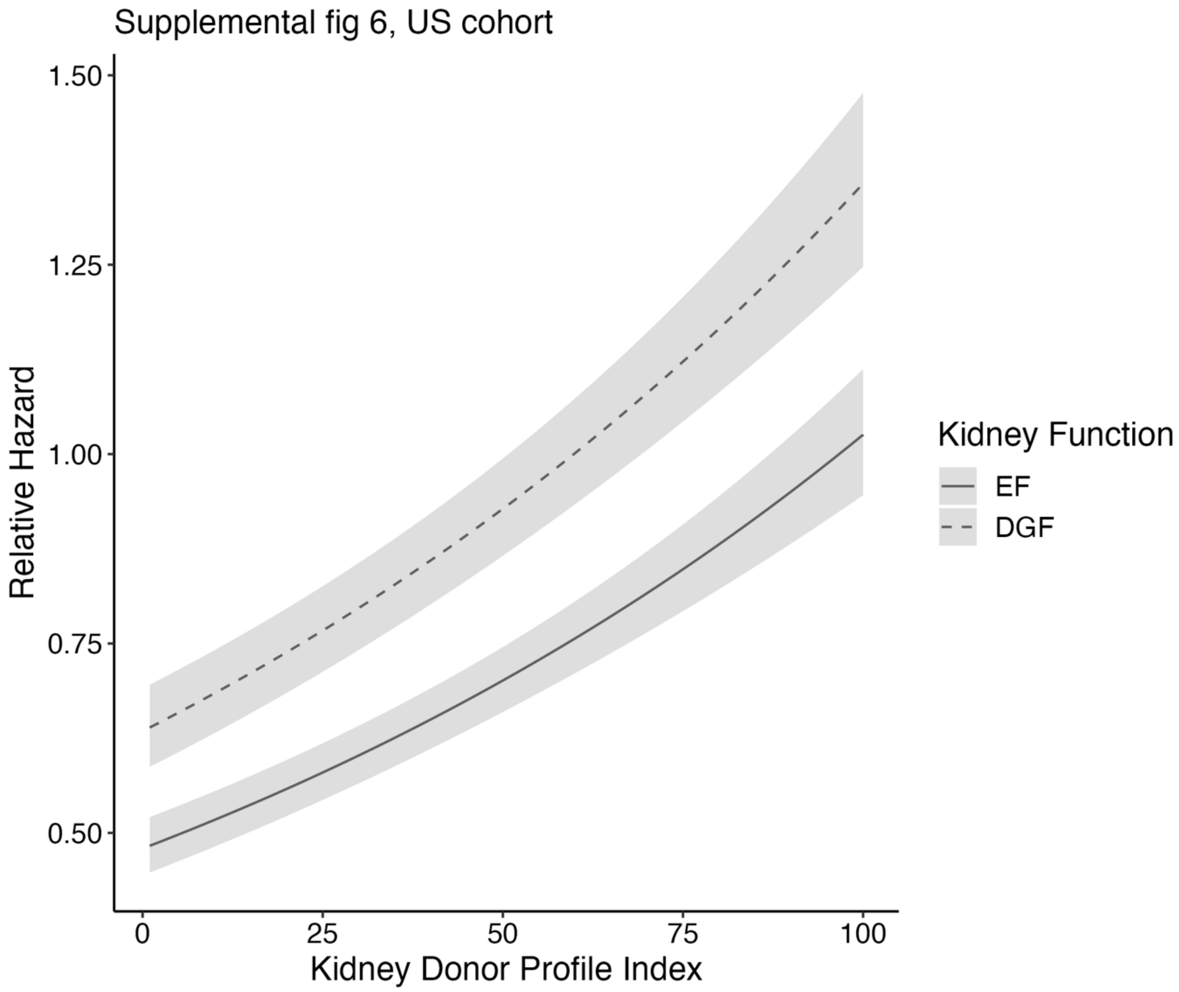

Supplemental figure 6

Restricted cubic spline analysis of kidney donor profile index as well as the association of kidney function, from 1 year and forward, in the US cohort. DGF is portrayed by the dashed line and EF by the solid line. The model is adjusted to CIT, recipient age, sex, recipient diabetes, machine perfusion, previous kidney transplant.

CIT = cold ischemia time, KT = kidney transplant, DGF = delayed graft function, EF = early function

Supplemental figure 7

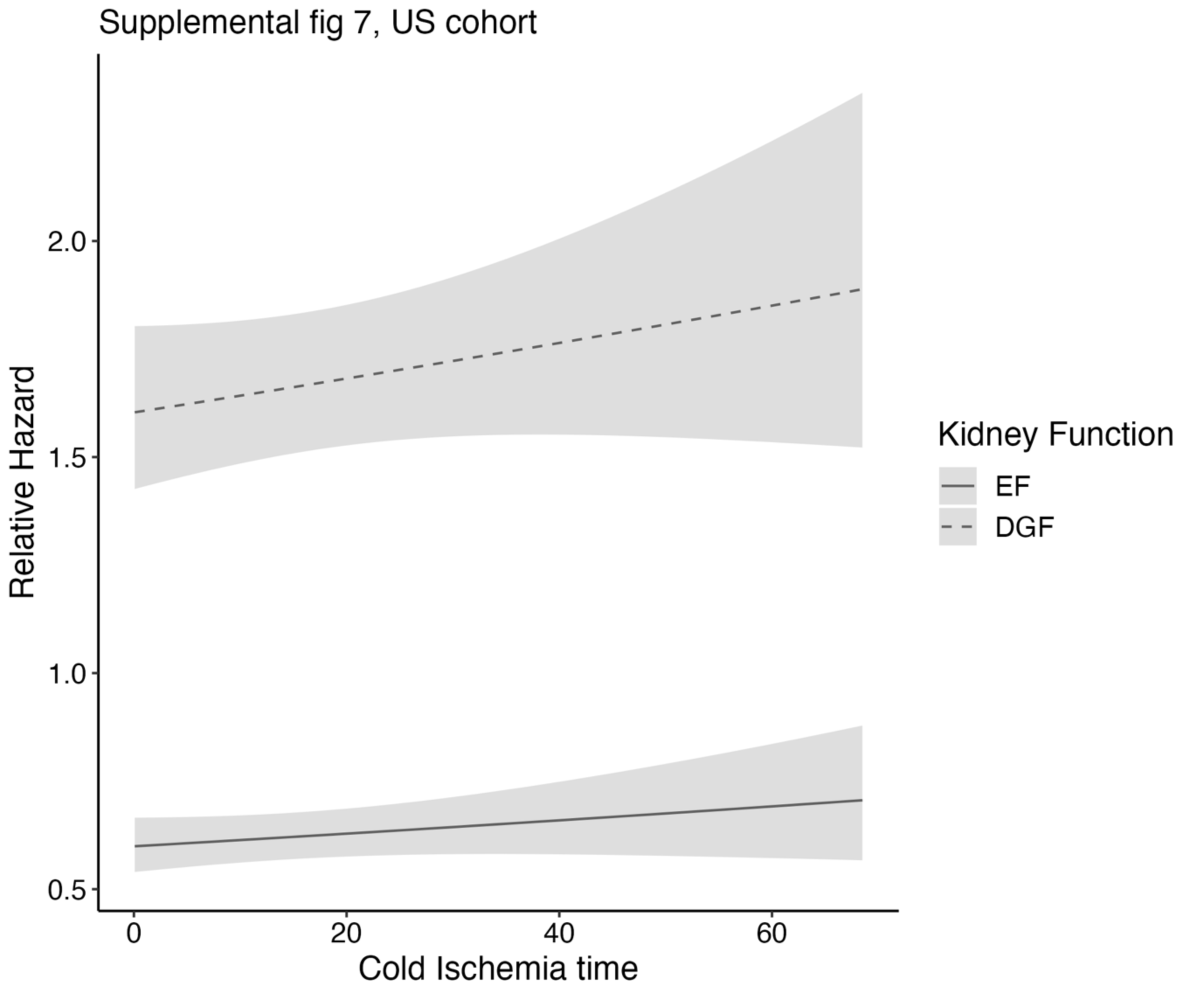

Supplemental figure 7
Restricted cubic spline analysis of cold ischemia time as well as the association of kidney function, during the first follow up year, in the US cohort. DGF is portrayed by the dashed line and EF by the solid line. The model is adjusted to CIT, recipient age, sex, recipient diabetes, machine perfusion, previous kidney transplant.
CIT = cold ischemia time, KT = kidney transplant, DGF = delayed graft function, EF = early function

Supplemental figure 8

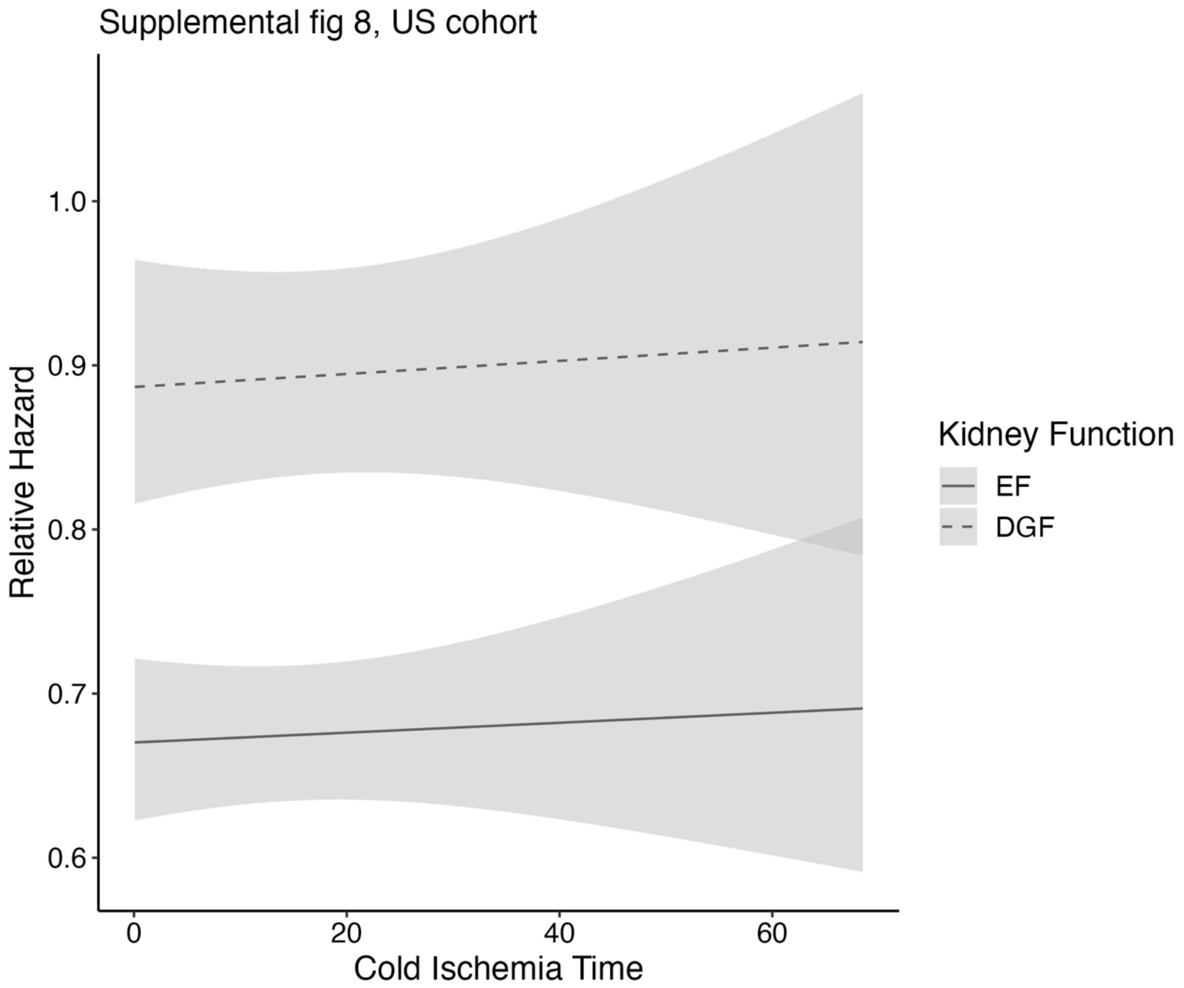

Supplemental figure 8
Restricted cubic spline analysis of cold ischemia time as well as the association of kidney function, from 1 year and forward, in the US cohort. DGF is portrayed by the dashed line and EF by the solid line. The model is adjusted to CIT, recipient age, sex, recipient diabetes, machine perfusion, previous kidney transplant.
CIT = cold ischemia time, KT = kidney transplant, DGF = delayed graft function, EF = early function

Supplemental tables

Supplemental table 1

Supplemental table 1 Survival table Finnish cohort

**Survival table Finnish cohort**		
**Outcome**	**EF (KM estimates)**	**DGF**
1 yr survival	97%	96%
3 yr survival	92%	89%

10 yr survival	66%		51%	
**Outcome**	**EF + CIT< 18hrs**	**EF + CIT ≥ 18hrs**	**DGF + CIT< 18hrs**	**DGF + CIT ≥ 18hrs**
1 yr survival	97%	97%	96%	95%
3 yr survival	94%	91%	85%	90%
10 yr survival	68%	65%	50%	51%
**Outcome**	**EF + KDPI<85**	**EF + KDPI≥85**	**DGF + KDPI<85**	**DGF + KDPI≥85**
1 yr survival	97%	97%	96%	91%
3 yr survival	93%	91%	91%	79%
10 yr survival	67%	54%	54%	36%

Supplemental table 2

Supplemental table 2 Survival table US cohort

**Survival table US cohort**				
**Outcome**	**EF (KM estimates)**		**DGF**	
1 yr survival	96%		89%	
3 yr survival	88%		79%	
**Outcome**	**EF + CIT< 16hrs**	**EF + CIT ≥ 16hrs**	**DGF + CIT< 16hrs**	**DGF + CIT ≥ 16hrs**
1 yr survival	97%	96%	89%	89%
3 yr survival	89%	88%	80%	78%
**Outcome**	**EF + KDPI<85**	**EF + KDPI≥85**	**DGF + KDPI<85**	**DGF + KDPI≥85**
1 yr survival	96%	90%	90%	84%
3 yr survival	89%	80%	81%	68%

Supplemental table 3

Supplemental table 3 Multivariable cox regression of time to death censored graft loss, Finnish cohort

Multivariable cox regression results for time to death censored graft loss, FIN cohort
(N = 2637)

**Characteristic**	**HR[1]**	**95% CI[1]**	**p-value**
Delayed graft function	1.35	1.06, 1.71	0.014
Cold ischemia time (per hour)	1.01	0.99, 1.04	0.38
Kidney donor profile index	Not applicable		0.01[2]
Recipient diabetes	1.28	0.99, 1.65	0.06
Recipient age (per year)	0.98	0.97, 0.99	<0.001

Recipient sex (male)	0.85	0.67, 1.08	0.19
Recipient peak PRA over 30%	1.17	0.83, 1.64	0.38
Previous kidney transplant	1.53	1.04, 2.25	0.032

[1]HR = Hazard Ratio, CI = Confidence interval
[2]p-value for non-linearity
PRA = Panel reactive antibodies

Supplemental table 4

Supplemental table 4 Multivariable cox regression of time to death censored graft loss, US cohort

Multivariable cox regression results for time to death censored graft loss, US cohort
(N = 60,919)

**Characteristic**	**HR[1]**	**95% CI[1]**	**p-value**
Delayed graft function	2.03	1.92, 2.16	< 0.001
Cold ischemia time (per hour)	Not applicable		0.03[2]
Kidney donor profile index	Not applicable		<0.001[2]
Recipient diabetes	1.05	0.98, 1.12	0.18
Recipient age (per year)	Not applicable		<0.001[2]
Recipient sex (male)	1.00	0.94, 1.06	0.96
Machine perfusion	1.07	1.00, 1.13	0.04
Previous kidney transplant	1.09	1.00, 1.18	0.047

[1]HR = Hazard Ratio, CI = Confidence interval
[2]p-value for non-linearity
